# Silver doped copper oxide decorated graphene oxide nanocomposite for electrochemical sensing, photocatalysis and biomedical applications

**DOI:** 10.1039/d5ra07285a

**Published:** 2025-11-11

**Authors:** Asmaa M. Alghamdi, Abid Ali

**Affiliations:** a Faculty of Science, Department of Biology, Al-Baha University Al-Baha Saudi Arabia; b Department of Chemistry, The University of Lahore 1-Km Defence Road Lahore 54590 Pakistan abid.ali@chem.uol.edu.pk

## Abstract

A silver-doped copper oxide nanocomposite decorated on graphene oxide (Ag–CuO@GO) was synthesized *via* a facile wet-chemical method and characterized using XRD, SEM, FTIR, UV-vis, and EDX analyses. The synergistic combination of Ag and CuO onto GO provided greater charge separation, better conductivity, and a high surface-area framework for uniform nanostructure distribution. Electrochemical studies confirmed excellent sensing performance toward dopamine, with rapid electron transfer kinetics and a low limit of detection (23.6 μM) and higher sensitivity of 3.43 mA cm^−2^ mM^−1^. Correspondingly, photocatalytic studies revealed that Ag–CuO@GO showed remarkable dye degradation capability. Ag–CuO@GO achieved up to 96% degradation of MB under direct sunlight with 0.3 mg mL^−1^ catalyst loading in 30 minutes. While kinetic studies at 0.2 mg mL^−1^ followed pseudo-first-order kinetics with an apparent rate constant of 0.019 min^−1^. Additionally, due to the intrinsic biomedical properties of Ag and CuO, the nanocomposite also exhibits notable antibacterial activity, further broadening its potential practical utility. These results confirm Ag–CuO@GO as an efficient multi-functional material for electrochemical sensing, photocatalytic degradation of organic dyes and antibacterial activity.

## Abstract 2

This study reports the fabrication of nanocomposite (Ag–CuO/GO), exhibits enhanced electrochemical sensing performance for biomolecule detection, superior photocatalytic degradation of organic pollutants, and notable antimicrobial activity.

## Graphical abstract

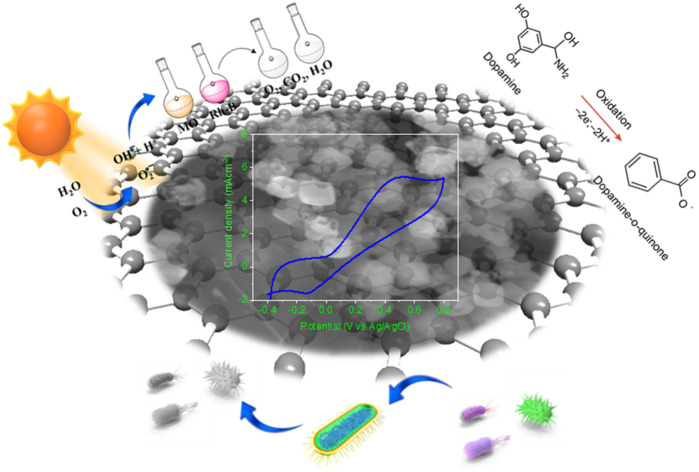

## 1 Introduction

The emergence of multidimensional nanomaterials has significantly advanced the development of multifunctional platforms for biomedical, environmental, electrochemical and photocatalytic applications.^1,2^ Among these, hybrid nanocomposites incorporating metal oxides, noble metals,^3^ where carbon-based supports have demonstrated exceptional physicochemical and functional properties.^4^ Copper oxide (CuO), a p-type semiconductor with a narrow band gap (∼1.2–1.9 eV), has garnered attention for its inherent redox activity, catalytic efficiency, cost-effectiveness, and biocompatibility.^5,6^ However, its practical use is often constrained by limited surface area, agglomeration, and suboptimal charge separation.^7^ To overcome these drawbacks, the integration of CuO with other functional components is essential.^8^

Doping with silver nanoparticles (Ag NPs) has proven to be a powerful strategy to boost CuO's photocatalytic and bactericidal activity.^9^ Owing to their strong surface plasmon resonance (SPR), electrical conductivity, and ion-releasing behavior, Ag NPs impart superior charge separation and antimicrobial efficiency when coupled with CuO.^10,11^ Additionally, graphene oxide (GO), a highly oxidized derivative of graphene, offers a large surface area, abundant oxygenated functional groups, and excellent dispersibility in aqueous media.^12^ GO not only enhances the mechanical stability and dispersion of metal/metal oxide nanoparticles but also promotes interfacial charge transfer, making it an ideal scaffold for nanohybrid architectures.^13,14^ In this context, the design of an Ag-doped CuO nanostructure anchored onto graphene oxide (GO) sheets (Ag–CuO@GO) presents a promising route to developing multifunctional nanomaterials.^15^ Such composites are expected to exhibit enhanced antimicrobial properties due to the synergistic interplay between Ag^+^-induced membrane disruption, CuO-mediated oxidative stress, and GO's ability to trap and anchor microbial cells.^11,16^ In the context of photocatalytic application, Ag–CuO@GO nanostructure could enhance the photocatalytic activity by facilitating efficient charge separation and transfer to and fro the electrolyte and electrode, where Ag acts as an electron sink, CuO absorbs visible light, and GO provides a high-surface-area conductive support.^17^ Moreover, the hybrid framework offers an electroactive surface suitable for the sensitive detection of biologically relevant analytes, such as dopamine (DA),^18^ a key neurotransmitter involved in numerous physiological processes.^19,20^

Dopamine is an essential neurotransmitter that requires accurate detection because aberrant levels are associated with neurological conditions including schizophrenia and Parkinson's. Because of its redox activity, it is perfect for electrochemical sensing with Ag–CuO@GO and other nanostructured materials.^21^ Electrochemical sensing platforms based on nanostructured electrodes offer rapid, selective, and low-cost solutions for real-time monitoring of biomolecules.^22^ For ultrasensitive sulfadimethoxine detection, a ternary Z-scheme/type-II heterojunction based on BiOCl/BiOIO_3_/BiOI allowed for improved charge separation and PEC response. A strong basis for food safety monitoring in intricate samples is provided by this design.^23^ Methylcellulose hydrogels supplemented with GO@Fe_3_O_4_ demonstrated improved electron transitions and energy band gap tuning, as well as improved optical and antibacterial capabilities. These multipurpose nanocomposites show promise for use in optoelectronic and biological applications of the future.^24^ The integration of Ag–CuO along with GO can further amplify the sensing response through efficient electron transfer and high catalytic activity.^25^ Recent studies have demonstrated the individual potential of Ag,^26^ CuO,^27^ and GO in biosensing and antibacterial applications;^28,29^ however, reports on their ternary hybrid systems with multi functionality remain limited.^30^

The present work addresses this gap by synthesizing a novel Ag–CuO@GO nanocomposite through a facile co-precipitation route followed by thermal treatment.^31^ The structural and morphological features were comprehensively characterized using XRD, SEM, EDX, FTIR, and UV-vis techniques. Electrochemical behavior of Ag–CuO@GO was evaluated *via*, cyclic and square wave voltammetry along with chronoamperometric studies using dopamine as a model analyte, while photocatalytic activity was assessed using MB dye. Moreover, its antibacterial performance was assessed against *E. coli* and *S. aureus* using disc diffusion assays. This study not only highlights the importance of material design in enhancing electrochemical transduction but also establishes a clear correlation between nanoscale architecture and antimicrobial efficacy. The combination of Ag doping, CuO semi-conductivity, and GO support confers the nanocomposite with superior physicochemical stability, rapid electron kinetics, and broad-spectrum antimicrobial action. These results suggest that the Ag–CuO@GO composite holds substantial promise as a multi-function material for future deployment in bioanalytical sensors, antimicrobial coatings, and water treatment technologies.

## 2 Experimental section

### 2.1 Materials

Graphene, copper chloride dihydrate (CuCl_2_·2H_2_O), silver chloride (AgCl) and trisodium citrate (Na_3_C_6_H_5_O_7_) were obtained from Sigma-Aldrich and used without further purification. Distilled water was employed throughout all solution preparations. Sodium hydroxide (NaOH) and ethanol were purchased from Merck. GAMRY 1010E potentiostat/galvanostat equipped with a three-electrode system-comprising a glassy carbon working electrode, platinum wire counter electrode, and Ag/AgCl (3 M KCl) reference electrode was used for all electrochemical measurements. All glassware was acid-washed and rinsed thoroughly with distilled water before use.

### 2.2 Synthesis of Ag–CuO@GO nanocomposite

Hydrophobic graphene was functionalized *via* acidic treatment to convert it into its hydrophilic graphene oxide (GO). Sulfuric acid (5 M) was used in chemical treatment to oxidize graphene into graphene oxide. A standard co-precipitation procedure was used for the synthesis of the silver doped CuO nanoparticles in a regular shape over graphene oxides nanosheets. Briefly, a suspension of 30 mg of GO was dispersed into 50 mL of deionized water under constant stirring. In a separate container 0.1 M (0.85 g) of CuCl_2_·2H_2_O and 5 mM (35.75 mg) of AgCl was dissolved in 50 mL of deionized water and stir the mixture until a clear solution was achieved. The resulting solution was gradually added to the suspension of GO under constantly stirring. In the next step, trisodium citrate (Na_3_C_6_H_5_O_7_) solution (50 mM) has been added in the above mixture and maintain its pH 10 *via* NaOH (2 M) solution added drop by drop. The system was constantly agitated for 10 hours at 60 °C and stayed overnight (24 hours) for the growth of well-ordered structure of silver doped copper oxide over the graphene oxide nanosheets. Finally, the product was collected and washed with ethanol and water several time and dried in an over at 50 °C.

### 2.3 Characterization

X-ray diffraction (XRD) analysis was performed using a Bruker D8 Advance Twin–Twin (Cu K) to examine the structural characteristics of Ag–CuO@GO NCs. The surface morphology was observed using scanning electron microscopy (SEM, Quanta FEG-250) to validate the nanostructure formation. Energy-dispersive X-ray spectroscopy (EDX) elemental mapping was performed for elemental confirmation, composition and distribution of Ag, Cu, O, and C. Fourier-transform infrared spectroscopy (FTIR, Bruker Alpha-II, ATR mode) was used to confirm the functional groups present on the surface of the NCs within the range of 4000–500 cm^−1^.

### 2.4 Electrochemical studies

Cyclic voltammetry (CV) was performed to evaluate the electrocatalytic performance of the synthesized Ag–CuO@GO nanocomposite. Electrochemical studies were conducted in phosphate buffer solution (PBS, pH 7.0) containing dopamine as the target analyte. A standard three-electrode setup was utilized in electrochemical studies to examine the redox behavior of nanocomposite, while measurements were recorded using a potentiostat/galvanostat (GAMRY 1010E) with the modified glassy carbon electrode (GCE) serving as the working electrode, Ag/AgCl as the reference, and platinum wire as the counter electrode. The CV curves were recorded at varying scan rates to assess the electron transfer kinetics and determine the diffusion-controlled behavior of dopamine oxidation. The glassy carbon electrode (GCE) was polished with alumina slurry followed by rinsed with distilled water as well as ethanol and dried at room temperature. A catalyst ink was prepared by dispersing 0.2 mg of Ag–CuO@GO in ethanol with Nafion as a binder under ultrasonication. Finally, drop casting method applied for the suspension of aliquot onto the GCE surface and dried to obtain the developed electrode (working) for electrochemical studies. All measurements were carried out at room temperature, and the electrode surface was cleaned and polished before each experiment.

### 2.5 Antibacterial activity assay

The antibacterial activity of Ag–CuO@GO was evaluated against *Escherichia coli* (ATCC 25922) and *Staphylococcus aureus* (ATCC 29213) by applying disk diffusion method. Bacterial suspensions were adjusted to 0.5 McFarland standard and were swabbed homogeneously onto Mueller–Hinton agar plates. Sterile filter paper disks of 6 mm diameter were infused with 5 μL and 10 μL of the Ag–CuO@GO composite solution (1 mg mL^−1^) and placed onto the inoculated agar. Disks infused with 10 μL of distilled water and streptomycin (1 mg mL^−1^) served as negative and positive controls, respectively. The plates were incubated at 37 °C for 24 hours, after which the zones of inhibition were measured. All experiments were performed in triplicate.

### 2.6 Photocatalytic degradation

The photocatalytic activity of Ag–CuO@GO was assessed using methylene blue (MB) dye as a model pollutant under direct sun light irradiation. 20 mg of the nanocomposite was dispersed in 100 mL of 5 ppm MB solution to maintain catalyst concentration of 0.2 mg mL^−1^. The solution was exposed to a direct sunlight source. Aliquots (3 mL) were collected at regular intervals and centrifuged to remove nanocomposites. Additionally, catalyst concentration effect was studied by varying the catalyst concentration from 0.05 mg mL^−1^ to 0.3 mg mL^−1^ while maintaining dye concentration same. The residual dye concentration was monitored using UV-vis spectroscopy by measuring the absorbance at *λ*_max_ = 664 nm. The degradation efficiency and kinetics were calculated using the formulas:1
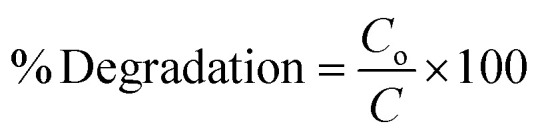
2
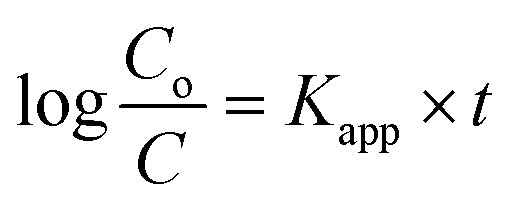
where *C*_o_ is initial concentration, *C* is concentration at time interval *t* and *K*_app_ is apparent rate constant. The stability of the Ag–CuO@GO composite was evaluated through five consecutive cycles of photocatalytic degradation MB using 0.3 mg mL^−1^ catalyst concentration. After each cycle, the catalyst was recovered by centrifugation at 8000 rpm for 5 minutes, washed thoroughly with deionized water and ethanol, and dried at 80 °C for 1 hour. The recovered catalyst was then reused in a fresh MB solution with the same initial concentration. The degradation efficiency for each cycle was calculated to assess the stability of the catalyst.

## 3 Results and discussion

### 3.1 Characterization of Ag–CuO@GO composite

#### 3.1.1 SEM

**Fig. 1:**
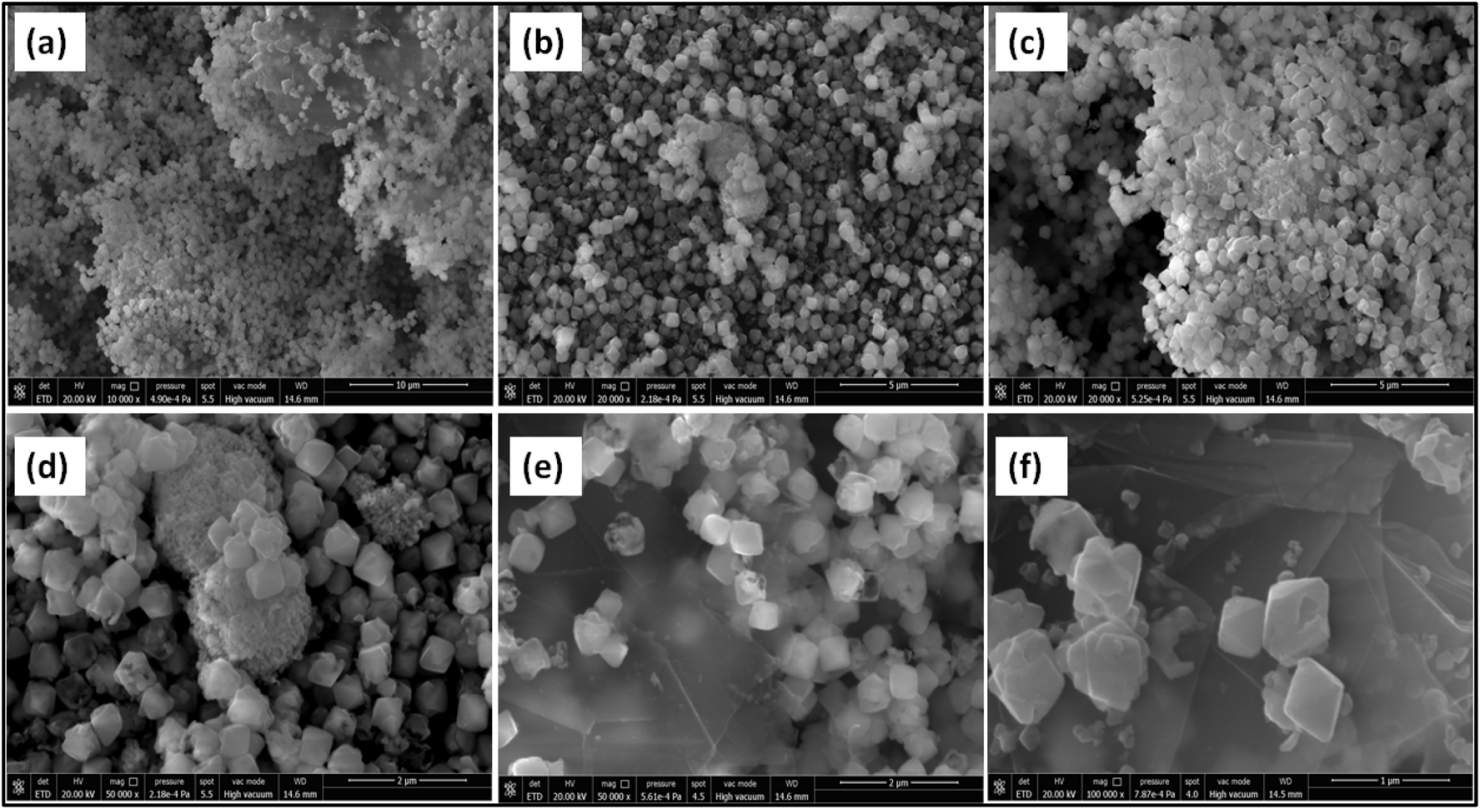
SEM images for the morphologies of Ag doped CuO@GO with (a–c) lower and (d–f) higher resolution.

Fig. 1 illustrates the surface morphologies of the synthesized nanomaterials captured through scanning electron microscopy (SEM) at varying magnifications. Images (a–c) correspond to pristine low-resolution image of CuO nanocubes, where Fig. 1(a) at 10 μm reveals agglomerated nanostructures forming a sponge-like matrix, suggesting a high surface-to-volume ratio beneficial for surface reactions. At higher magnification in Fig. 1(b and c) at 5 μm scale, respectively, the CuO nanoparticles exhibit a denser distribution and more defined granular boundaries, indicating improved crystallinity and uniform growth. In contrast, Fig. 1(d–f) displays the morphological evolution following Ag doping and GO integration with high resolution. In (d) and (e), the emergence of cubic and faceted structures is observed, characteristic of Ag-decorated CuO with enhanced crystalline domains. Notably, Fig. 1(f) captured at 1 μm magnification reveals a heterogeneous visuality, where sharp-edged nano cubes are embedded over layered graphene oxide (GO) sheets.^32^ This structural arrangement confirms the successful anchoring of Ag–CuO onto GO, offering a favorable hybrid architecture for electrochemical activity due to increased active sites and improved electron transport pathways.

#### 3.1.2 EDX

**Fig. 2:**
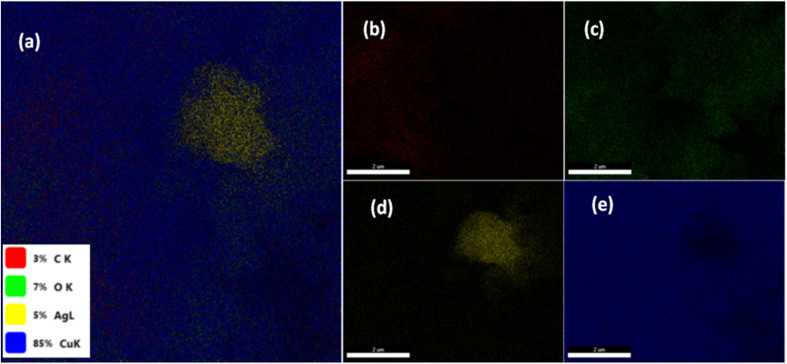
EDX elemental mapping of silver doped CuO@GO composite. (a) Survey analysis (b) carbon (c) oxygen (d) silver and (e) copper.

Energy-dispersive X-ray spectroscopy (EDX) was employed to confirm the elemental composition and spatial distribution of the components in the prepared material.^33^ EDX mapping provides localized elemental information, validating the presence and uniform dispersion of key elements throughout the sample surface. Fig. 2(a–e) displays the elemental mapping images obtained *via* energy-dispersive X-ray spectroscopy (EDX), revealing the uniform distribution of elements within the Ag–CuO@GO nanocomposite. The survey analysis in Fig. 2(a) confirms a well-integrated structure, with overlapping signals corresponding to the constituent elements. Fig. 2(b) shows the distribution of carbon, characterized by red coloration, while Fig. 2(c) represents oxygen in green, indicating the presence of the graphene oxide (GO) support. Fig. 2(d) reveals silver as dopant, distributed marked in yellow, affirming the presence of silver as dopant while Fig. 2(e) depicts the copper as most abundant within the fabricated hybrid (Ag–CuO@GO). Elemental distribution is validating the dispersion of each element across the observed area. The mapping data collectively confirms the successful decoration of doped CuO nanoparticles on the GO matrix with homogeneous elemental dispersion, substantiating the composite's morphological and compositional integrity.

#### 3.1.3 XRD

**Fig. 3:**
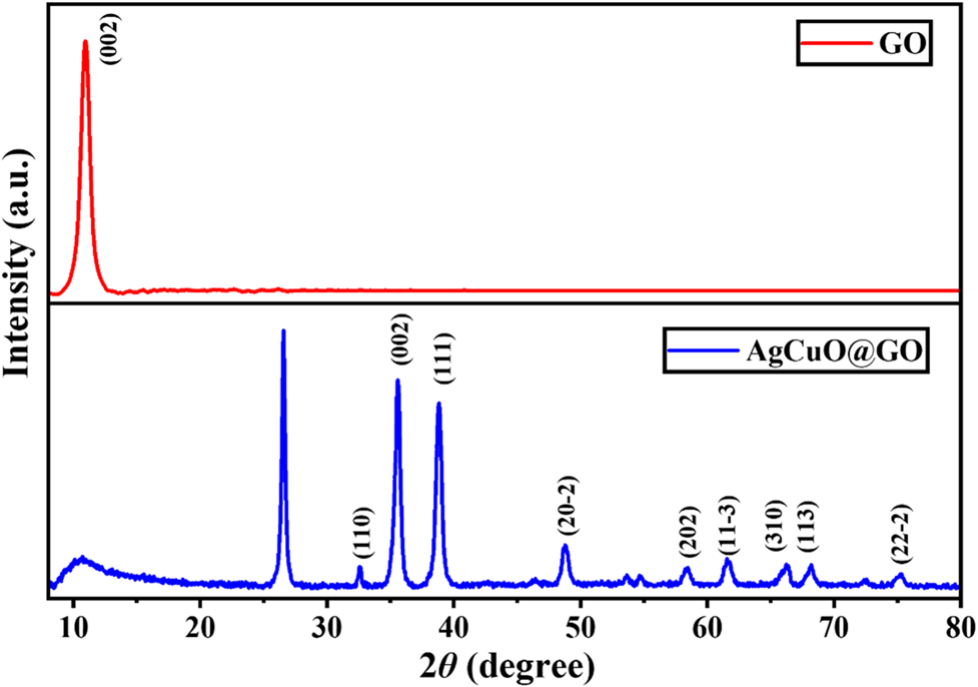
XRD patterns of graphene oxide (GO) and Ag-doped CuO@GO showing monoclinic CuO phases.

The crystalline integrity and phase identification of the synthesized nanomaterial were investigated using X-ray diffraction analysis.^34^ As depicted in Fig. 3, the diffractogram of Ag-doped CuO exhibits well-defined peaks, indexed to the monoclinic phase of CuO (JCPDS no. 80-1916),^35^ with additional minor reflections corresponding to metallic Ag (JCPDS no. 04-0783),^36^ confirming successful incorporation of silver into the copper oxide lattice. The broad diffraction hump centered around 10.9° observed in the GO sample is characteristic of its amorphous layered structure with oxygenated functionalities.^37^ Upon hybridization with Ag–CuO, this peak diminishes significantly, indicating a strong interfacial interaction between the GO sheet and the metal oxide nanoparticles. The crystallite size of Ag–CuO was estimated using the Debye–Scherrer formula, yielding average dimensions in the nanometric regime (∼18–25 nm),^38^ indicative of a high surface-to-volume ratio beneficial for photocatalytic and microbial interactions. Moreover, these outcomes validate the successful structural integration of Ag and CuO on the GO matrix, laying the foundation for enhanced charge mobility and antimicrobial performance.

#### 3.1.4 FTIR

**Fig. 4:**
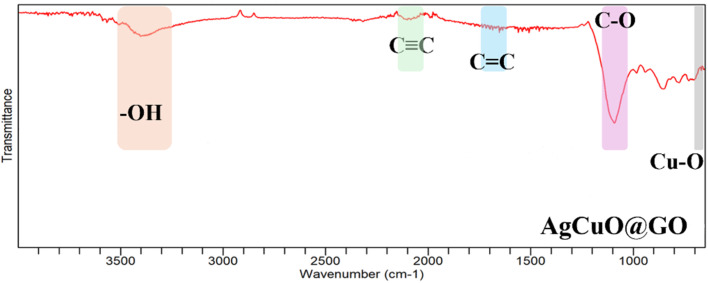
FTIR spectra of Ag-doped CuO@GO.

Fig. 4 illustrates the Fourier-transform infrared (FTIR) spectra of the synthesized silver doped CuO decorated at graphene oxide (CuO@GO) nanocomposite, spanning the spectral range of 4000–500 cm^−1^. The GO spectrum (blue trace) displays a series of well-defined peaks indicative of abundant oxygen-containing functional groups that arise due to the oxidative treatment of graphite during the GO synthesis. A broad absorption band centered around 3400 cm^−1^ corresponds to the O–H stretching vibrations of hydroxyl groups and intercalated water molecules.^39^ The peak near 2130 cm^−1^ can be attributed to the C≡C stretching vibrations from residual hydrocarbons.

The prominent band at 1615 cm^−1^ is associated with the skeletal C=C stretching vibrations of the unoxidized graphitic domains or the adsorbed water's H–O–H bending mode. In addition, the peak around 1080 cm^−1^ is assignable to the alkoxy C–O stretching vibrations, reinforcing the presence of oxygenated moieties across GO sheets.^40^

Notably, absorption bands emerge in the below 700 cm^−1^ region. These are attributed to the Cu–O stretching vibrations, confirming the incorporation of copper oxide nanoparticles.^41^ The presence of these vibrations substantiates the successful loading and chemical anchoring of CuO species onto the GO surface. Additionally, the overall reduction in band intensities in the CuO@GO spectrum relative to GO not only reflects the removal of oxygen functionalities but also indicates a strong metal support interaction between CuO and the GO matrix, essential for enhancing the composite's physicochemical properties. Thus, it confirms the successful synthesis of CuO@GO by highlighting the chemical transformation of GO into a reduced form and the effective incorporation of CuO nanoparticles, making the composite suitable for potential applications in catalysis, sensing, or energy storage.

### 3.2 Photocatalytic studies

**Fig. 5:**
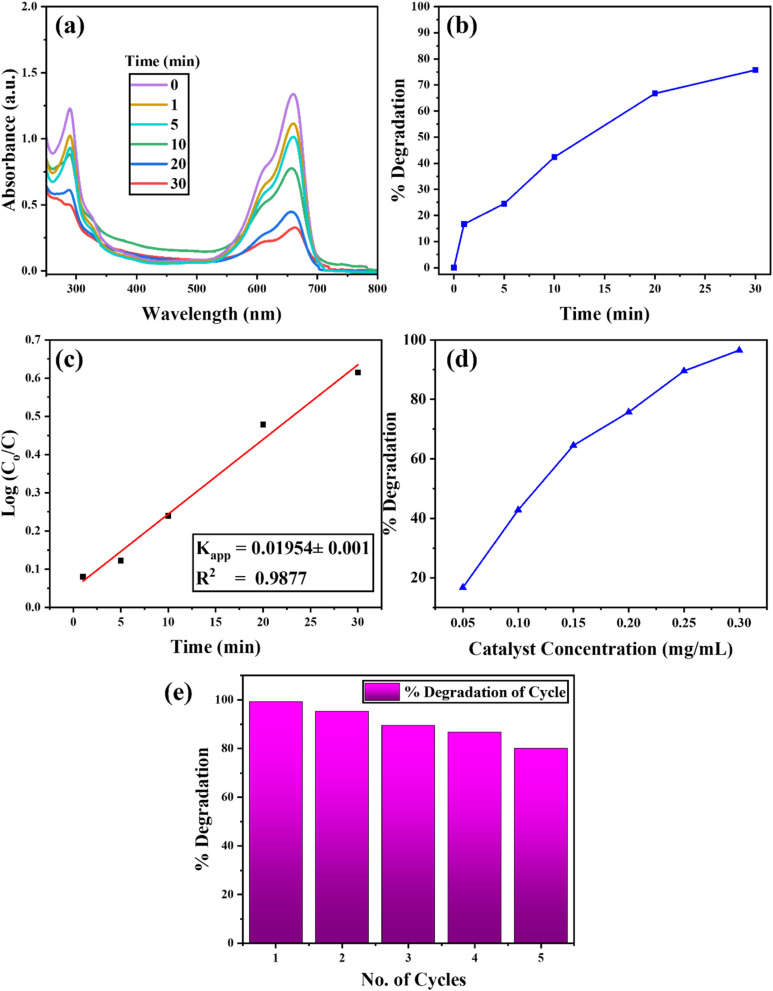
(a) UV-vis spectra showing time-dependent decrease in absorbance. (b) % degradation of the dye (c) apparent rate constant of dye degradation (d) effect of the concentration of catalyst (e) reusability studies of the catalyst against MB *via* consecutive cycles.

The photocatalytic activity of Ag–CuO@GO was assessed through the degradation of MB dye under visible light irradiation. As shown in Fig. 5(a–c), intensity of the absorption peak of MB at 664 nm decreased over time, demonstrating efficient degradation of the dye. The NCs showed 75.7% MB degradation within 30 minutes at a catalyst concentration of 0.2 mg mL^−1^. When the concentration was increased to 0.3 mg mL^−1^, the degradation efficiency rose up to 96%, as shown in Fig. 5(d), representing excellent photocatalytic performance of NCs. The degradation kinetics followed a pseudo-first-order model, with a calculated rate constant (*K*_app_) of 0.019 min^−1^, confirming rapid pollutant removal.

The higher performance is due to the synergistic effects of Ag, CuO, and GO. Ag NPs enhance visible light absorption *via* surface plasmon resonance (SPR), while CuO helps electron–hole pair generation. GO acts as an electron acceptor, reducing recombination and facilitating the generation of reactive oxygen species (ROS) such as ˙OH and O_2_˙^−^, which are responsible for oxidative degradation of MB molecules.^42^Fig. 5(e) presents the reusability of the Ag–CuO@GO composite for the degradation of MB dye in five consecutive cycles, indicating its stability. The observed decrease in degradation efficiency is due to the blockage of active sites by adsorbed intermediate species and the minimal mechanical loss of catalyst during the recovery process between cycles. The results highlight the potential of Ag–CuO@GO as a highly efficient photocatalyst for environmental applications.

### 3.3 Electrochemical sensing

#### 3.3.1 Electrochemical response of DA on Ag–CuO@GO electrode

**Fig. 6:**
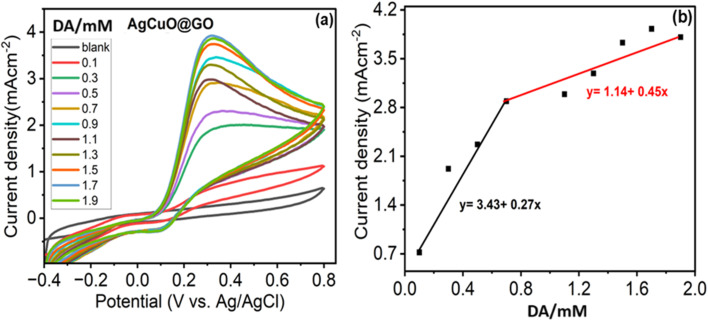
(a) Cyclic voltammogram for Ag–CuO@GO modified at the glassy carbon electrode (GCE) electrode with different concentration of dopamine in phosphate buffer solution (PBS) and (b) corresponding linear calibration plot.

Fig. 6(a) illustrates the cyclic voltammetry (CV) response of the fabricated composite electrode in the presence of increasing concentrations of dopamine (DA), ranging from 0 mM to 1.1 mM in 0.2 mM intervals. A distinct anodic peak appears near +0.36 V, corresponding to the oxidation of dopamine to dopamine-*o*-quinone. The cathodic peak observed around 0 V represents the reverse reduction process, indicating a quasi-reversible redox couple. As the dopamine concentration increases, the redox peak currents gradually increase, demonstrating the excellent electrocatalytic activity of the composite material toward dopamine oxidation. The enhanced electrochemical performance can be attributed to the synergistic combination of silver and copper oxide nanoparticles uniformly anchored on the graphene oxide matrix. The CuO offers redox-active sites, while Ag nanoparticles contribute to improved electrical conductivity and enhanced electron mobility. The presence of graphene oxide ensures a high surface area, favoring increased dopamine adsorption and electron exchange.^43^ The integration of these components falls into a porous and conductive framework, promoting effective redox interaction with dopamine. As shown in the calibration plot derived from Fig. 6(b), a linear range of 0 to 1.1 mM was established for dopamine detection, with a regression coefficient of (*R*^2^ = 0.98) confirming a strong linear correlation, also enabling the electrode to be suitable for the quantitative determination of dopamine in similar ranges. The limit of detection (LOD) was determined to be 23.6 μM, and the limit of quantification (LOQ) was calculated as 71 μM, respectively. Furthermore, the sensitivity of the sensor was calculated to be 3.43 mA cm^−2^ mM^−1^·, indicating the system's ability to produce a strong electrochemical response per unit concentration per electrode area. The linearity also suggests diffusion-controlled behavior, implying that the dopamine molecules diffuse toward the electrode surface and undergo efficient electron transfer upon interaction with the sensing material.

**Fig. 7:**
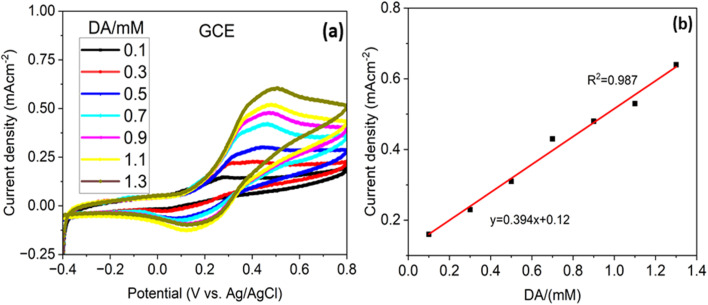
(a) Cyclic voltammogram for bare GCE electrode with different concentration of dopamine in PBS and (b) its linear calibration plot.

The high sensitivity of the Ag–CuO@GO electrode reflects its superior catalytic activity and efficient electron transfer at the analyte–electrode interface. In comparison with bare glassy carbon electrode (GCE) as shown in the Fig. 7, the sensitivity is far behind with the value of 0.394 mA cm^−2^ mM^−1^ reflected from the calibration curves in Fig. 7(b). The reproducible and stable CV response underscores its reliability for dopamine detection.

Beyond sensing, the same composite's redox-active components and surface reactivity also contribute to its antimicrobial efficacy, highlighting its dual-functional potential in both bio-electrochemical diagnostics and microbial inhibition.

**Fig. 8:**
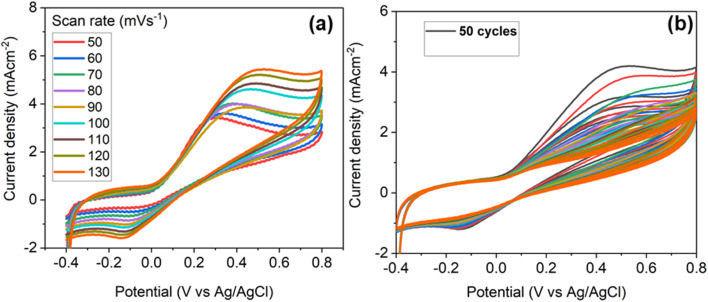
Cyclic voltammograms with (a) different scan rates (50–130 mV s^−1^) at Ag–CuO@GO modified GCE under fixed concentration and (b) CV cycles (50) for the stability test with fixed concentration and scan rate.

A diffusion-controlled electrochemical process is indicated by the cyclic voltammetry (CV) plots in Fig. 8(a), which shows that the current density at the Ag–CuO@GCE electrode increases as the scan rate (SR) rises from 50 to 130 mV s^−1^. This implies that the charge transfer is improved at faster scan rates because of the increased flux of electroactive species towards the electrode surface and that the redox reaction kinetics are comparatively quick. Capacitive behavior with faradaic contributions is further implied by the quasi-reversible nature of the redox peaks moving with scan rate. The Ag–CuO nanostructure's surface reconfiguration or electrochemical activation is indicated by the steady decline in current density seen in the CV curves over fifty consecutive cycles in Fig. 8(b). This investigation showed that the fabricated composite electrode could be used for several times to assess the analyte including glucose and related biomolecules.

This improvement is probably the result of enhanced electrolyte penetration, greater electron conductivity and improved electroactive surface area all of which work together to gradually expose catalytically active areas improving the electrode's redox performance.

The data presented in Table 1 substantiate the electrochemical competence of the Ag–CuO@GO sensor toward electrochemical detection of dopamine. The low LOD signifies the sensor's capacity to detect trace dopamine levels, essential for accurate monitoring in biological or clinical matrices. The wide linear range enables detection across physiological dopamine concentrations, while the high sensitivity affirms the material's catalytic strength. Overall, these metrics validate the electrode's performance and highlight its prospective use in advanced electrochemical sensing applications.

**Table 1:** Electrochemical performance parameters for dopamine detection using Ag–CuO@GO electrode

Electrode	Analyte	Sensitivity (mA cm^−2^ mM^−1^)	LOD (μM)	LOQ (μM)	LR (mM)
Ag–CuO@GO	Dopamine	3.43	23.6	71	0.1–0.7
					0.7–1.9
GCE	Dopamine	0.394	124.7	372	0.1–1.3

#### 3.3.2 Chronoamperometric response

**Fig. 9:**
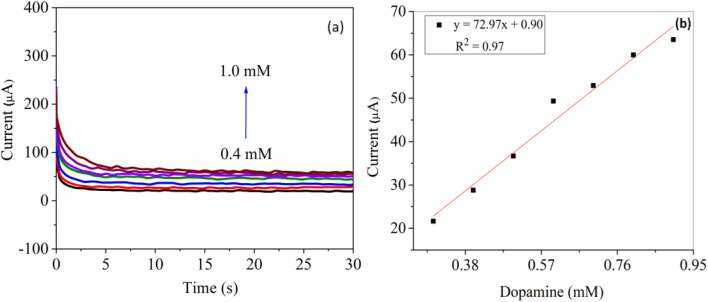
(a) Chronoamperometric response of Ag–CuO@GO electrode for dopamine (0.4–1.0 mM) under the redox potential applied and (b) its linear calibration plot.

Fig. 9(a and b) present the chronoamperometric response and corresponding calibration behavior of the Ag–CuO@GO modified electrode for dopamine (DA) detection under successive additions of the analyte. As shown in Fig. 8(a), each incremental addition of dopamine (ranging from 0.4 mM to 1.0 mM) induces a distinct and immediate increase in current, followed by gradual stabilization, consistent with a diffusion-controlled oxidation process. The well-resolved and reproducible current highlights the rapid electron transfer kinetics and strong electrocatalytic interaction between dopamine and the nanocomposite surface. The corresponding calibration curve in Fig. 9(b) demonstrates a clear linear relationship between the steady-state current and dopamine concentration, with a regression equation I (μA) = 72.97C + 0.90 and a correlation coefficient (*R*^2^ = 0.97). This linearity confirms the electrode's quantitative sensing capability across the tested range (0.4–1.0 mM), further substantiated by its low detection and quantification with a higher sensitivity (72.97 μA cm^−2^ mM^−1^ for chrono). The chronoamperometric data affirm that the Ag–CuO@GO electrode exhibits reliable, fast-response behavior and high analytical performance, positioning it as a promising platform for real-time neurotransmitter monitoring in complex media.

**Fig. 10:**
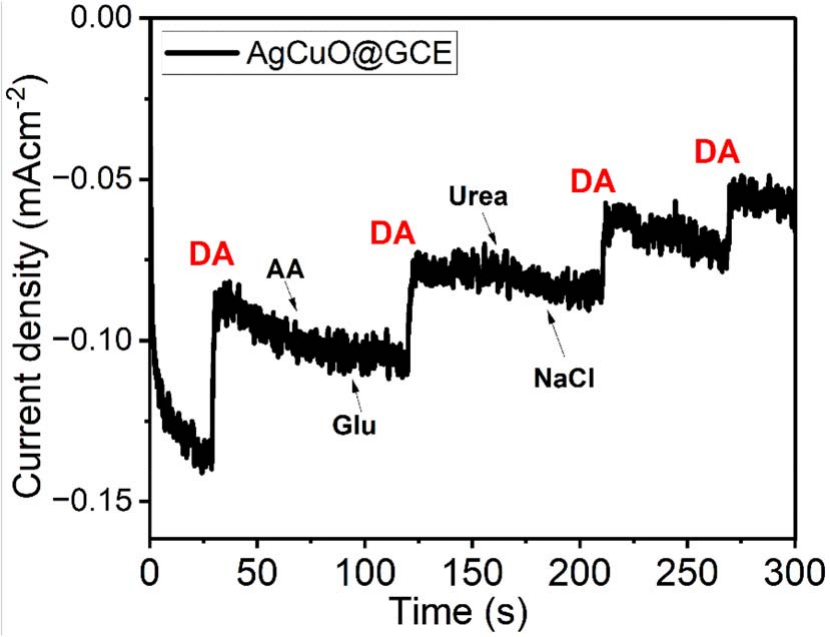
Staircase voltammogram at the Ag-doped CuO@GO modified GCE electrode exhibiting the effect of interference study.

High selectivity and stability are demonstrated by the Ag–CuO@GCE electrode's amperometry response to dopamine (DA) in the presence of common interfering species, such as ascorbic acid (AA), glucose (Glu), urea, and NaCl (Fig. 10). As a result of DA's distinct catechol structure and particular adsorption behavior, the electro-oxidation of DA is selectively facilitated by the synergistic redox activity and surface chemistry of Ag and CuO. Despite being electroactive, AA and Glu generate much lower current responses most likely as a result of kinetic impediment, electrostatic repulsion or weak contact with the electrode surface. Due to their electrochemical inactivity at the specified voltage, urea and NaCl produce very small current changes indicating very little interference. The electrode's great affinity and good anti-interference performance-essential characteristics for selective DA sensing in biological environments are highlighted by the noticeable and rapid increase in current that occurs with each reintroduction of DA.

#### 3.3.3 Square wave voltammetry

**Fig. 11:**
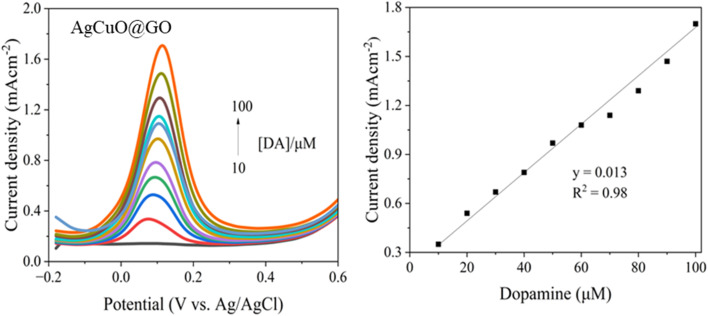
Square wave voltammograms of AgCuO@GO modified GCE electrode toward dopamine detection at varying concentration, and (b) the corresponding calibration plot.

The electrochemical performance of AgCuO@GO modified GCE electrode toward dopamine detection was evaluated using square wave voltammetry (SWV) as demonstrated in Fig. 11(a). The SWV profile exhibit a distinct oxidation peak at 0.12 V (*vs.* Ag/AgCl) which corresponds to electrochemical oxidation of dopamine. With increasing DA concentration from 10 to 100 μM a progressive enhancement in peak current density is observed, indicating a strong concentration dependent response. The corresponding calibration curve (Fig. 11(b)) demonstrates a linear relationship between the peak current density and DA concentration, expressed by the equation *y* = 0.013× with correlation coefficient (*R*^2^) of 0.98 signifying excellent linearity. The sensitivity of electrode is reports as 13 mA cm^−2^ μM^−1^, additionally the LOD is 6 μM reflecting the lowest concentration of dopamine that can be accurately detected using this sensor.

### 3.4 Antimicrobial activity

The antibacterial activity of Ag–CuO@GO nanocomposite demonstrated in Table 2 against both *E. coli* and *S. aureus*, with a clear response. The larger zones of inhibition observed at higher loadings of 10 μL suggest that the efficacy of nanocomposite is influenced by concentration. This is due to increased availability of ROS. The greater exposure of *E. coli* compared to *S. aureus* may be due to structural differences in their cell membrane. *E. coli*, with its thinner peptidoglycan layer and outer membrane, may allow easier interaction with ROS, whereas the thick, cross-linked peptidoglycan layer of *S. aureus* provides a stronger barrier,^44^ necessitating higher concentration of ROS for effective disruption.

**Table 2:** Antibacterial activity *via* disc diffusion method using Ag–CuO@GO nanocomposite

Sample	Loading (1 mg mL^−1^)	Zone of inhibition	
		*E. coli*	*S. aureus*
Ag–CuO@GO	5 μL	18 ± 0.1 mm	12 ± 0.1 mm
	10 μL	20 ± 0.1 mm	17 ± 0.1 mm
Streptomycin	10 μL	40 ± 0.1 mm	40 ± 0.1 mm

The antimicrobial mechanism of Ag–CuO@GO is based on the ability to generate ROS. These oxidative agents induce severe cellular damage, such as lipid peroxidation, protein oxidation, and DNA strand breaks, leading to cell death. The GO matrix enhances this activity by helping electron transfer and stabilizing the metal nanoparticles for ROS generation. Although Ag–CuO@GO showed strong antibacterial activity, it was less active than the standard streptomycin. However, the ROS facilitated mechanism make Ag–CuO@GO as a valuable candidate against antibiotic-resistant strains, where conventional antibiotics may fail. Future work should focus on optimizing the synthesis to enhance ROS yield, evaluating biocompatibility, and exploring synergistic effects with existing antibiotics to maximize its therapeutic potential.

## 4. Conclusion

In this study, an Ag–CuO nanocomposite decorated on graphene oxide (Ag–CuO@GO) was effectively synthesized *via* a wet-chemical method and comprehensively characterized using multiple analytical techniques. The synergistic effect of Ag, CuO, and GO offered enhanced charge separation, conductivity, and a high-surface-area for effective electron transfer. Electrochemical studies verified excellent sensing performance for dopamine with quick response and low limit of detection, confirming applicability of Ag–CuO@GO in biosensing. In addition, the nanocomposite revealed notable photocatalytic activity, attaining efficient MB degradation under direct sunlight. Ag–CuO@GO showed tunable degradation efficiency by changing catalyst concentration and following pseudo-first-order kinetics. In addition to electrochemical and photocatalytic methods, the Ag–CuO to GO composite showed strong antibacterial features, which is attributed to the inherent biocidal nature of silver and copper(ii) oxide, which increases its potential real-world applications. Combined, these findings make Ag–CuO and GO a highly multifunctional nanomaterial with significant possibilities in electrochemical sensing, environmental remediation settings, as well as with its antibacterial benefits. While demonstrating the efficacy of the Ag–CuO@GO composite, this study is limited to a single Ag doping concentration and the use of GO as the sole carbon support. Future work should involve a systematic variation of Ag dopant levels and extend to other carbon matrices like rGO, CNTs, and carbon nano-onions to identify the most synergistic and active catalyst configuration for multifunctional activity.

## Conflicts of interest

There are no conflict to declare.

## Data Availability

All relevant data supporting the findings of this study are included in the article. Additional datasets generated and/or analyzed during the current study are available from the corresponding author upon reasonable request.
